# Connective tissue and genetics in the pathogenesis of rectal prolapse: A scoping review

**DOI:** 10.1111/codi.70628

**Published:** 2026-09-21

**Authors:** Matthew Davenport, Alexander O'Connor, William G. Newman, Andrew P. Morris, Abhiram Sharma, Gemma Faulkner, Dipesh H. Vasant, John McLaughlin, Edward Kiff, Karen Telford

**Affiliations:** ^1^ Faculty of Biology, Medicine, and Health The University of Manchester Manchester UK; ^2^ Department of Colorectal Surgery Manchester University NHS Foundation Trust Manchester UK; ^3^ Manchester Centre for Genomic Medicine Manchester University NHS Foundation Trust Manchester UK; ^4^ Neurogastroenterology Unit, Gastroenterology, Wythenshawe Hospital Manchester University NHS Foundation Trust Manchester UK; ^5^ Department of Gastroenterology, Salford Royal Hospital Northern Care Alliance NHS Foundation Trust Salford UK

**Keywords:** collagen, connective tissue, connective tissue disorder, elastin, genetic association, genetic testing, joint hypermobility, rectal prolapse

## Abstract

**Aim:**

Rectal prolapse can occur in individuals who lack the established risk factors (constipation, obstetric trauma and advanced age). A recognised association with hereditary connective tissue diseases may indicate a role for variants in connective tissue‐related genes in the pathogenesis of the disease. This scoping review evaluates the evidence supporting this.

**Methods:**

A scoping review was performed using a systematic search of Medline, Embase, CINAHL and The Cochrane Library in March 2026. Studies describing any of the following in the context of rectal prolapse were included: epidemiology in individuals with hereditary connective tissue disease, tissue morphology, disease heritability or genetic testing of affected individuals. Study design and key findings were collated and summarised.

**Results:**

Sixty of 4442 screened sources were eligible for inclusion. Rectal prolapse is common in Ehlers‐Danlos Syndrome (3.2%–18%), Marfan's syndrome (6%) and joint hypermobility (4.9%–11%). Hypermobility is associated with post‐operative recurrence and younger age of onset. Abnormal pelvic floor collagen and elastin organisation, reduced myofibroblast density and increased dermal elastin density were key examples of abnormal tissue morphology in individuals with rectal prolapse. Patterns of heritability have not been adequately evaluated, but novel variants in connective tissue‐related genes (e.g. *EFEMP1, COLGALT2, FBLN5* and *TNXB)* are reported in individuals with rectal prolapse.

**Conclusion:**

An association with hereditary connective tissue disease, and the presence of abnormal morphology of the extracellular matrix characterise the relationship between connective tissue and rectal prolapse. There is evidence to support a genetic association with rectal prolapse, though further characterisation of heritability and larger scale association studies are required.

**What does this paper add to the literature?**

This is the first systematic evaluation of the evidence regarding the genetic contribution to the risk of developing rectal prolapse. This review pulls together evidence from diverse methodologies regarding the role of defective or deficient connective tissues in the pathogenesis of the disease, and the evidence for a genetic aetiology.

## INTRODUCTION

External rectal prolapse (ERP) is a condition in which the rectal wall folds in on itself and protrudes through the anus [1]. It is relatively rare, with European studies reporting an incidence of 0.003%–0.02% [2, 3]. ERP most commonly affects older women [2, 3], and key risk factors include obstetric trauma and age‐related weakening of the pelvic floor.

However, rectal prolapse is not limited to this group. It also occurs in younger patients (6%–16% in individuals under 40 years) [2, 4, 5], in nulliparous women (approximately 30% of cases) [6] and in men (accounting for over 50% of cases in some Asian cohorts) [7, 8, 9, 10]. Constipation and straining are also important risk factors, although they do not fully explain the condition, as only a small proportion of individuals with constipation develop rectal prolapse [11].

There is evidence linking rectal prolapse with joint hypermobility [12] and hereditary disorders of connective tissue (HDCT), such as Ehlers‐Danlos syndrome (EDS) [13]. This has led to the suggestion that a genetic predisposition may play a role, potentially involving genes involved in connective tissue synthesis and maintenance.

The primary aim of this scoping review was to identify and describe the evidence supporting a genetic contribution to rectal prolapse due to variants in connective tissue‐related genes. A scoping review was chosen due to the infancy of the literature on this topic, and the anticipated diverse range of methodologies used. Although there is a substantial body of research regarding the genetic basis of the related condition of pelvic organ prolapse (POP) (affecting the anterior and middle pelvic compartments) [14, 15], the evidence for rectal prolapse is less well established. To date, no systematic reviews have addressed this question.

## METHODS

### Study design

This scoping review was conducted in line with the JBI guidelines [16], and reported according to the PRISMA‐ScR (PRISMA Extension for scoping reviews) checklist [17]. The protocol was registered prospectively on Open Science Framework (DOI: 10.17605/OSF.IO/ND5B7).

### Search strategy

The search strategy was designed in collaboration with a medical librarian. A broad spectrum of search terms was chosen to identify sources across a range of disciplines and methodologies. Searches were carried out in October 2025 (and updated in March 2026) on Medline, Embase, CINAHL and the Cochrane Library with results limited to those available in English, but with no publication date restrictions. The full search strategy is shown in supplementary Figure S1.

### Eligibility criteria

Due to the anticipated scarcity of data in this field, published abstracts were included in addition to peer‐reviewed studies. Relevant review articles were included during full‐text screening to ensure that any additional primary sources of data cited in these reviews were identified.

**FIGURE 1:**
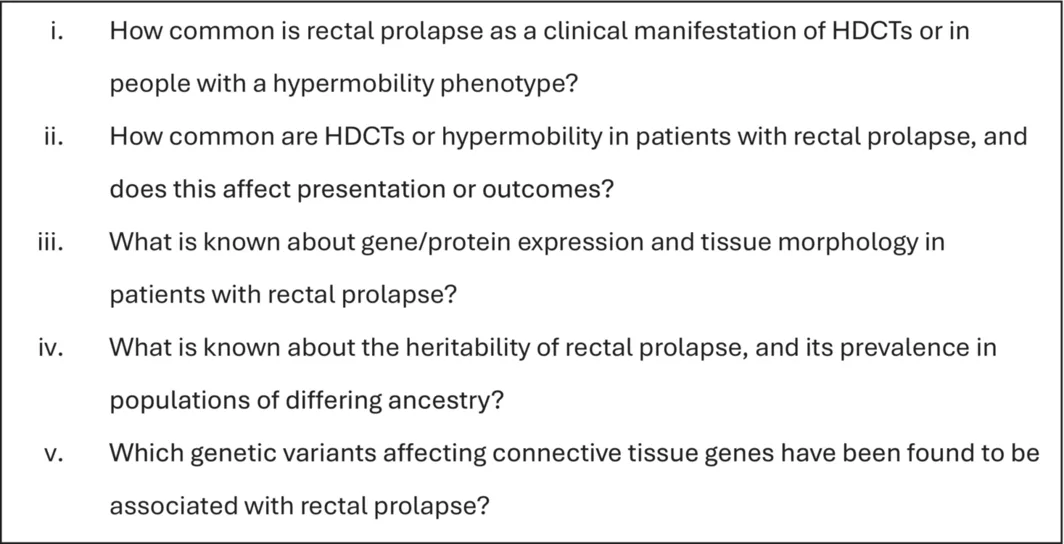
Predetermined questions to be addressed in this scoping review, which were used to develop a list of search terms for literature search.

Search terms were chosen to ensure each of the five sub‐questions listed in Figure 1 were addressed. Addressing the central question of this scoping review required identification of both evidence regarding the role of defective or deficient connective tissue in the aetiology of rectal prolapse and that there was a proposed genetic cause for this.

Sources describing genetic connective tissue defects in the closely related condition of POP were intentionally sought in the initial search. These manuscripts were used to identify high quality contemporary evidence to provide additional insight in the discussion section. These sources were not, however, eligible for inclusion in the scoping review itself.

### Screening, data charting and data analysis

After excluding duplicates, two investigators (MD and AO) performed title and abstract screening. Screening was conducted using review software Rayan (Cambridge, MA, USA). Sources that addressed any of the five sub‐questions in Figure 1 were included for full‐text screening. Sources were excluded if not addressing any sub‐question. This incorporated studies that solely described data from patients with POP or conditions other than rectal prolapse, studies purely describing surgical techniques or novel devices in ERP, and animal studies.

Full‐text review and data charting was performed using a database in Microsoft Excel (Redmond, WA, USA). Recorded data charting variables are shown in supplementary Figure S2. Reference lists of included studies were reviewed to identify additional relevant sources. Due to the heterogeneity of the literature, a narrative synthesis of the results is presented.

## RESULTS

**FIGURE 2:**
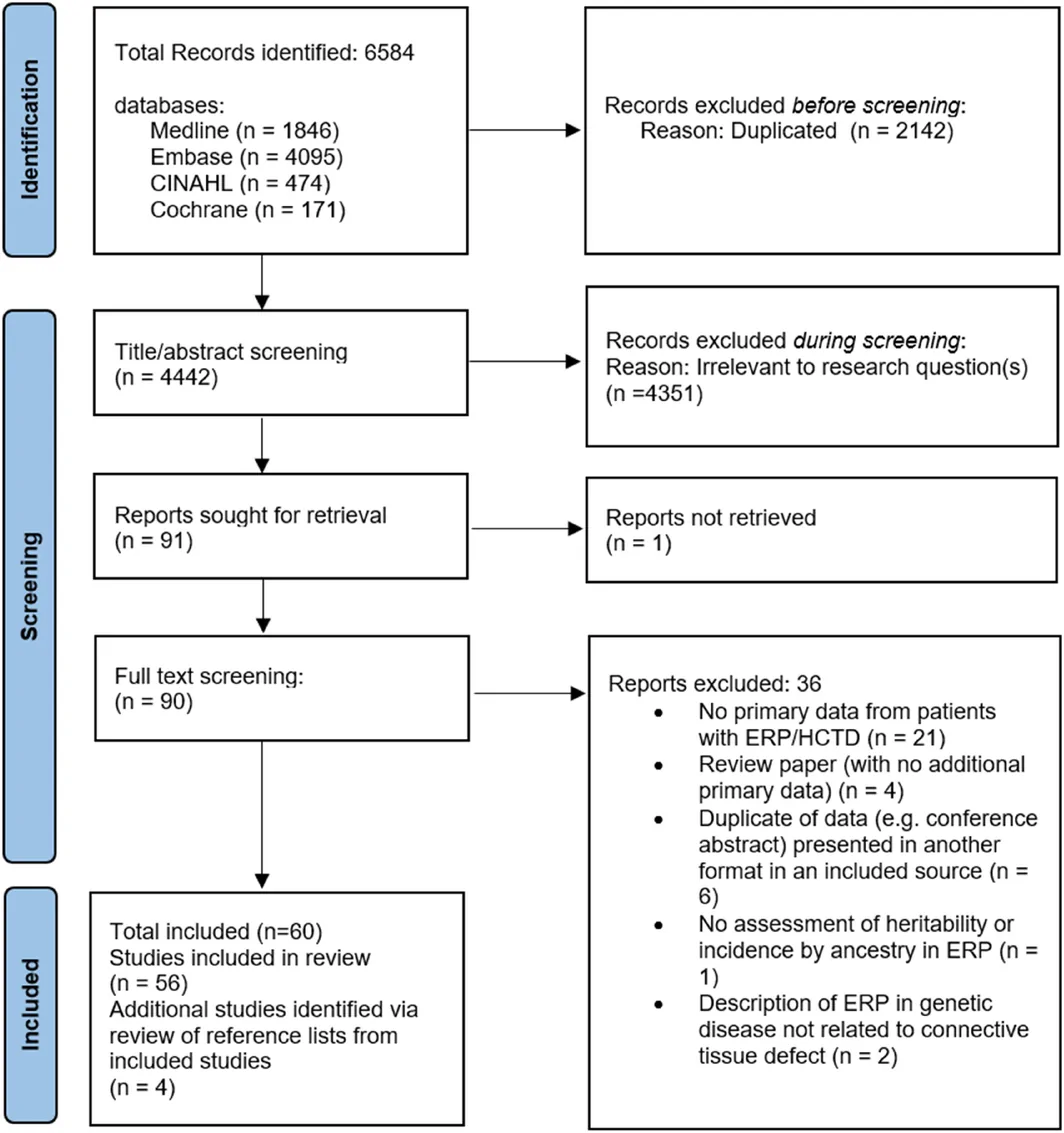
PRISMA flow chart demonstrating results of literature search, screening and source selection process.

A total of 6584 sources were identified. After screening, 60 sources were selected for inclusion in the review. A PRISMA flow chart is shown in Figure 2. The results are described below in reference to five pre‐defined sub‐questions listed in Figure 1.

### How common is rectal prolapse as a clinical manifestation in HDCT or in people with a hypermobility phenotype?

Eighteen resources, including 16 peer‐reviewed publications and 2 published abstracts, reported the incidence of rectal prolapse in patients with a HDCT. All but three studies (all case reports) were based in North America or Western Europe. The study characteristics and outcomes are shown in Table 1.

**TABLE 1:** Characteristics and key findings of studies of rectal prolapse in patients with HDCTs or hypermobility.

Study	Study design	Country	Hereditary disorder of connective tissue	Population characteristics	Incidence of rectal prolapse	Control group	Incidence of rectal prolapse in control group
Beighton [20]	Cross‐sectional	USA	EDS	Adults with EDS (not distinguished by subtype) *n = 125*	3.2%		
Boechler [27]	Case–control	USA	William's Syndrome^†^	Williams Syndrome (all ages) *n = 62*	24.2%*	Healthy controls *n = 36*	2.8%
Castori [26]	Cross‐sectional	Italy	EDS	Post‐partum women with EDS *n = 82*	11.1%		
Darakjian [21]	Case–control	USA	Hypermobility	Adults with Hypermobile EDS *n = 423*	8.5%*	Adults without hypermobility or HDCT *n = 55*	0%
				Adults with Hypermobility spectrum disorder (HSD) *(defined by 2017 international diagnostic criteria)* [83] *n* = 1389	4.9%*		
				Hypermobile adult controls *(defined as patients with historical HSD or localised joint hypermobility, not meeting 2017 international diagnostic criteria for HSD or hEDS)* [83] *n* = 221	5%		
Demirdas [24]	Cross‐sectional	Netherlands	EDS	Classic‐like EDS (all ages) *n = 17*	17.6%		
Frank [22]	Cross‐sectional	France	EDS	Adults with Vascular EDS *n = 133*	0.75%		
Green [23]	Cross‐sectional	UK	EDS	classic‐like EDS (all ages) *n = 22*	27%		
Janmey [25]	Case–control	USA	EDS	Gynaecological pelvic floor clinic attendees with EDS *n = 40*	15%*	Gynaecological pelvic floor clinic attendees without connective tissue disease *n* = 164	2.4%
Kciuk [19]	Cross‐sectional	Canada	EDS	Adult females with EDS *n = 1303*	18%		
Lam [34]	Cross‐sectional	UK	Hypermobility	Adults with hypermobility on intestinal failure units *(‘hypermobility disorders’ not specifically defined)* *n* = 84	2%		
Loganathan [33]	Cross‐sectional	USA	Hypermobility	Adults with ‘Joint Hypermobility Syndrome’ (JHS) *(defined as meeting the Beighton Criteria threshold for JHS diagnosis)* [20] *n* = 27	11%		
Mohammed [35]	Case–control	UK	Hypermobility	Adults with Rectal evacuatory disorder and ‘Joint hypermobility’ (JHM) *(JMN defined as a score of ≥2/5 on validated hypermobility screening tool)* [84] *n* = 65	8%	Adults with rectal evacuatory disorder without joint hypermobility disorder *n* = 135	3%
Nee [18]	Cross‐sectional	USA	Marfan's Syndrome	Adults with Marfan's syndrome *n = 600*	6%		
			EDS	Adults with EDS *n* = 804	16.4%		
Case reports							
Study	Location	Condition	Genetic testing	Phenotype description			
Choudhary [29]	India	Congenital cutis Laxa	None	2 month old female, loose wrinkled and inelastic skin, bilateral inguinal hernias and multicompartment pelvic organ prolapse (inc rectal prolapse)			
Douglas [28]	Australia	EDS	None	2 year old male with joint hyperextensibility, inelastic skin and rectal prolapse			
Gold [32]	USA	Williams syndrome	Yes (1.5 Mb deletion at 7q11 encompassing ELN gene – diagnostic for Williams syndrome)	Developmental delay, rectal prolapse (at age 1), precocious puberty, hypertension and aortic coarctation, dysmorphic features (all in childhood)			
Sahn [30]	USA	Infantile systemic hyalinosis	6,XX, 15 ph + karyotype (normal)	Neonate with Flexion contractures, torticollis, oral/peri‐anal skin lesions, sclerodermatous skin and rectal prolapse			
Mirkheshti [31]	Iran	Osteogenesis imperfecta (type IV)	None	21 year old female with thoracic kyphosis, multiple previous lower extremity fractures, rectal prolapse			

* Statistically significant (*p* < 0.05) compared to listed comparator group.

^†^ Williams syndrome is not a ‘hereditary disorder of connective tissue’, but the responsible genetic mutation results in a deletion of 26–28 genes on chromosome 7, including the ELN gene (gene for elastin).

The prevalence of ERP in adults with EDS ranges from 3.2% to 18% [18, 19, 20], markedly higher than the general population [2, 3]. When differentiated by EDS subtype, prevalence in hypermobile, vascular and classic‐like EDS (clEDS) was 8.5% [21], 0.75% [22] and 17.6%–27% [23, 24] respectively. ERP was significantly more common in adults with EDS versus ‘non‐hypermobile controls’ (8.5% vs. 0%) [21] and in pelvic floor clinic attendees with EDS versus attendees without EDS (15% vs. 2.4%) [25]. In subgroups of patients with EDS, the incidence of ERP was 11.1% (post‐partum women) [26], and 15% (pelvic floor clinic attendees) [25]. In other conditions, the prevalence of ERP was 6% in Marfan's syndrome [18] and 24.2% in Williams syndrome [27]. ERP was also described in case reports as a trait in EDS [28], congenital cutis laxa [29], infantile systemic hyalinosis [30], osteogenesis imperfecta [31] and Williams Syndrome [32].

In adult patients with clinical features of joint hypermobility, the prevalence of ERP was 4.9%–11% [21, 33]. In subgroups with hypermobility, the prevalence of ERP was 2% in hypermobile adults on intestinal failure units [34] and the incidence was 8% in hypermobile adults with concurrent rectal evacuatory disorder (not significantly higher than in non‐hypermobile patients) [35]. The way in which hypermobility is defined in each of these studies is detailed in Table 1. The severity of perineal descent, which is proposed to be a precursor to rectal prolapse, showed no correlation with the degree of joint hypermobility (quantified by the Beighton score [36]) [37].

The co‐occurrence of rectal prolapse with surgical conditions in which impaired connective tissue function is proposed to contribute to the pathogenesis is also reported. In patients with diverticular disease the odds ratio of also having rectal prolapse was 3.9× higher than in those without [38]. In patients with POP, the prevalence of rectal prolapse was 2.1%–13% [39, 40], which was significantly higher than the rate of ERP in patients without POP in one study (13% vs. 0%) [39]. POP was also present in 29.9%–48% of patients with ERP [40, 41, 42], which was significantly higher than the incidence in those without [41, 42]. Furthermore, the co‐occurrence of POP was associated with a higher chance of rectal prolapse recurrence after surgery (52.4% vs. 28.6%) [43].

### How common are HDCTs or hypermobility in patients with rectal prolapse, and does this affect presentation or outcomes?

Five resources, including three peer‐reviewed manuscripts and two published abstracts reported the impact of joint hypermobility in rectal prolapse. The degree of joint mobility (quantified by maximum extent of fifth finger passive extension) was significantly greater in patients with ERP than in those without [12]. A diagnosis of a connective tissue disease [44] and the presence of generalised hypermobility (defined as a Beighton score ≥4) [45] are predictors of post‐operative recurrence in two studies. In a third study, a diagnosis of a ‘collagen vascular disease’ was not a predictor of recurrence [46]. Among patients with rectal prolapse, those with hypermobility (defined as meeting diagnostic criteria for ‘benign joint hypermobility syndrome’) were also significantly younger at diagnosis [45, 47]. A co‐existing connective tissue disease was significantly more common in patients with rectal prolapse, than in those with POP (0.7% vs. 0.42%) [40].

### What is known about gene/protein expression and tissue morphology in patients with rectal prolapse?

Nineteen resources, including 11 peer‐reviewed papers and nine published abstracts investigated immunohistochemical characteristics of tissues from patients with rectal prolapse. In 16 of these, findings were compared to a group of healthy subjects or to patients with an alternative pathology. Findings from these resources are summarised in Table 2.

**TABLE 2:** Characteristics and key findings from studies of tissue morphology and protein/gene expression in rectal prolapse.

Study	Country	Study design *(tissue)*	Patients, *n*	Comparator group, *n*	Tissue characteristic examined	Key findings
Bassotti [64]	Italy	Immunohistochemistry *(full thickness rectal biopsies)*	Patients with IRP/OD *n* = 17	Patients with rectal cancer *n* = 10	Distribution and abundance of cells of enteric nervous system and sex hormone receptors	IRP and OD associated with significant reduction in submucosal and myenteric plexus enteric glial cell & significant increase in ICCs Significantly fewer IRP/OD patients had β‐oestrogen receptors expressed on enteric neurons than controls
Cao [50]	China	Immunohistochemistry, staining and western blot *(surgical specimens)*	Patients with IRP/OD *n* = 56	Patients with Grade 3/4 haemorrhoids *n* = 43	MMP1, TIMP1, IL6 and TNFa expression and ECM fibre characteristics	IRP/OD associated with higher MMP1, IL6 and TNFa expression but lower TIMP1 expression Tissue from IRP/OD patients has thinner collagen fibres and disordered elastin fibres
Joshi [57]	UK	Histology with Orcein staining and mechanical testing with Zwick Roell device *(skin biopsies)*	Patients with rectal prolapse *n* = 20	Patients undergoing surgery for ‘other indication’ *n* = 21	Dermal elastin expression and skin biopsy tensile strength	Patients with rectal prolapse had significantly higher percentage of dermal elastin and greater Young's modulus (elasticity) than controls
Joshi [58]	UK	Immunohistochemistry *(skin biopsies)* and qPCR *(RNA from cultured fibroblasts)*	Patients with rectal prolapse *n* = 20	Patients undergoing surgery for ‘other indication’ *n* = 21	Fibulin‐5 staining intensity and mRNA expression	No significant difference in fibrulin‐5 staining intensity or mRNA expression between cases and controls. In subgroup analysis, young males patients had significantly reduced fibrulin‐5 staining intensity and mRNA expression versus young male controls
Kraemer [63]	Germany	Immunohistochemistry *(STARR procedure specimens)*	Patients with IRP *n* = 51		Histological characteristics of rectum in patients with IRP	Common characteristics: myenteric plexus fibrosis (85%), circular muscle layer fibrosis/atrophy (81/68%), longitudinal muscle layer fibrosis/atrophy (65/35%), moderate‐advanced ‘neuropathological defects (49%)
Kang [55]	UK	Histology and Van Gieson staining*(surgical specimens)*	Patient with ERP *n* = 11	Patients with SRUS (*n* = 13) or colorectal cancer (*n* = 9)	Morphology of rectal wall and collagen content	Rectal wall layer thickness similar in ERP and colorectal cancer, but thickness significantly higher than both in SRUS. Submucosal and muscularis propria collagen content higher in ERP versus controls, but higher than both in SRUS (no test of statistical significance)
Lonsdale [60]	UK	Histology and staining *(rectal biopsies)*	Patients with either ERP or mucosal prolapse *n* = 13		Microvascular changes in mucosa and submucosa in ‘mucosal prolapse’	Microvascular features: Mucosal vessels: ectasia (95%) erosions (55%) and muscularised capillaries (50%) Submucosal vessels: Endothelial vacuolation (36%) Medial hypertrophy (36%) and intimal hyperplasia (32%)
Oppitz [62]	Germany	Immunohistochemistry *(surgical specimens)*	Patients with rectal prolapse *n* = 46	‘Controls’ (not specified) *n* = 38	Morphology of submucosal plexus of ENS	No significance differences in neuronal density or giant ganglia distribution
Sileri [65]	Italy	Immunohistochemistry *(STARR procedure specimens)*	Patients undergoing STARR procedure for rectal prolapse *n* = 13	Patients undergoing rectal resection for ‘other indication’ *n* = 14	Distribution And density of ICCs	Topographic distribution of ICC was similar to controls, but significantly higher c‐Kit+ ICC density in distal rectal tissues of the rectal prolapse group
Smyth [48]	UK	Histology *(surgical specimens—pelvic floor tissue)*	Patients with ERP *n* = 18		Morphology of connective tissue in POD	Fascial projections running through the mesorectum blend continuously with the endopelvic fascia & collagenous layer beneath peritoneal reflection Elastic fibres are present both within the subperitoneal collagenous layer and running with the fascial projections towards the endopelvic fascia
Smyth [51]	UK	Immunohistochemistry *(surgical specimens—pelvic floor tissue)*	Patients with ERP *n* = 37	‘controls’ (not specified) *n* = 6	Proportion and distribution of myofibroblasts	Males and multiparous women with rectal prolapse have significantly reduced myofibroblasts in their pelvic connective tissues compared to controls
Smyth (2011b) [53]	UK	Histology and Van Gieson staining *(surgical specimens—pelvic floor tissue)*	Patient with ERP *n* = 33		Morphology of the subperitoneal collagen layer	Multiparous females had a significantly thicker collagen layer than males/nulliparous females
Smyth (2011c) [52]	UK	Immunohistochemistry *(surgical specimens—pelvic floor tissue)*	Patient with ERP *n = unspecified*	Patients with colonic cancer *n = unspecified*	Cellular proliferation activity	Cellular proliferation activity was significantly higher in females (nulliparous and parous) versus controls. Proliferation was also higher in male patients versus controls, but not statistically significant
Smyth [54]	UK	Immunohistochemistry *(surgical specimens—pelvic floor tissue)*	Patient with ERP *n* = 61	Patients with colorectal cancer *n* = 5	Distribution of collagen fibres	Collagenous bands anchoring rectum to endopelvic fascia contain collagen I and III (similar pattern and fibre distribution seen in controls) Collagenous bands were thinner in men/nulliparous women than in parous women (p value not stated)
Smyth (2011e) [59]	UK	Histology and Van Gieson staining *(surgical specimens—pelvic floor tissue)*	Patient with ERP *n* = 65	Patients with colorectal cancer *n* = 6	Cellularity, evidence of angiogenesis and presence of fibrous bands in perirectal tissues	Only tissue from multiparous female patients demonstrated significantly increased cellularity/presence of fibrous bands versus controls Patients demonstrated evidence of neovascularisation which was not seen in controls
Smyth [46]	UK	Immunohistochemistry *(surgical specimens—pelvic floor tissue)*	Patient with ERP *n* = 16	‘Controls’ (unspecified) *n* = 4	MMP1 expression (and its impact on collagenous band thickness)	Patients had increased amounts of MMP1 compared to controls (p value not stated). higher MMP1 expression associated with fewer collagenous bands supporting the rectum (p value not stated)
Smyth [47]	UK	Histology and Van Gieson staining (surgical specimens—pelvic floor tissue)	Patient with ERP *n* = 70	Patients with colorectal cancer *n* = 5	Integrity of collagen layer and elastin fibres, and presence of fat herniation	Disruption of collagen layer and elastic fibres, and fat herniation seen in patients but not controls (unclear if significant)
Warren [56]	UK	Histology and Van Gieson staining (pararectal tissue)	Patients with ‘mucosal rectal prolapse’ *n* = 32	Patients with IBS, adenomas, IBD or ischaemic colitis *n* = 118	Distinguishing histological characteristics	‘diamond‐shaped crypts’ and elastosis significantly more commonly seen in mucosal rectal prolapse than other anorectal pathologies
Zorenkov [61]	Germany	Histology (full thickness rectal biopsies)	Young males with rectal prolapse *n* = 5	Patients with colorectal cancer *n* = 15	Histological features of ENS	Patients had significantly higher mean ganglionic area, mean neuronal content of submucosal ganglia and frequency of submucosal ganglia containing ≥7 neurons

Abbreviations: ECM, extracellular matrix; ENS, enteric nervous system; ICC, interstitial cells of Cajal; IL6, interleukin‐6; IRP, internal rectal prolapse; MMP1, matrix metalloproteinase‐1; OD, obstructed defecation; POD, pouch of Douglas; SRUS, solitary rectal ulcer syndrome; STARR, stapled transanal rectal resection; TIMP1, tissue inhibitor of metalloproteinases‐1; TNFa, tumour necrosis factor alpha.

In a series of studies by a group in Oxford, UK, the histological characteristics of pelvic floor tissues in patients with ERP were compared with tissues from patients with other conditions (‘control group’). They reported disruption of normal collagen layer and elastin fibre organisation in patients with ERP [48, 49] that was not seen in the control group and higher matrix metalloproteinase 1 (MMP1) expression [50] (the latter finding was replicated by another study, in patients with rectal intussusception (RI) [51]). RI refers to the invagination and caudal protrusion of the rectal wall into the distal rectum or anal canal but not beyond the anal verge and may be a precursor to ERP. The Oxford group also demonstrated that male and parous female patients with ERP had reduced connective tissue myofibroblast density [52], whereas only females had higher myofibroblast proliferative activity compared with controls [53]. Males and nulliparous females with ERP had a thinner subperitoneal collagenous layer and thinner collagenous bands anchoring the rectum to the endopelvic fascia than parous women with ERP [54, 55], but the relative concentration of Type I and III collagen in these collagenous bands was similar to the control group. Other authors have reported that ‘excess’ rectal wall collagen content is frequently seen in ERP but never in controls [56], whereas mucosal elastosis is significantly associated with mucosal prolapse [57].

To investigate whether connective tissue changes in patients with rectal prolapse are a systemic phenomenon (rather than a secondary effect of tissue trauma), histological examination of skin biopsies has been utilised. Patients with ERP had a higher percentage of dermal elastin and skin samples had greater elasticity on mechanical testing than samples from patients without ERP [58]. Furthermore, young male patients with ERP had significantly reduced dermal fibulin‐5 staining intensity and mRNA expression compared to age and gender‐matched controls [59].

Additional studies describe other characteristics of tissue morphology including neovascularisation [60, 61] in both patients with ERP and mucosal prolapse, and an increase in the cellularity of the enteric nervous system (ENS) within the rectal wall in young males with rectal prolapse [62]. However, in a further study, ENS neuronal density and giant ganglia distribution matched those seen in patients without prolapse [63].

Further characterisation of the enteric nervous system is described in studies of tissues from patients with RI. Rectal biopsies from patients with RI showed deleterious changes to ENS architecture (including myenteric plexus fibrosis and reduction in enteric glial cell density) [64, 65]. Conversely, the density of interstitial cells of Cajal was either similar [66] or increased in patients with RI compared to those without [65]. These conflicting findings fail to provide a clear illustration of the pattern of defects of the ENS. Such changes may also demonstrate a secondary effect of RI, rather than pre‐existing susceptibility to its development.

### What is known about the heritability of rectal prolapse, and its prevalence in populations of differing ancestry?

No familial aggregation or formal twin studies were identified, with only a single report of rectal prolapse occurring in male twins in adulthood in the literature [67]. In one study, where ethnicity data were reported in 43 patients, there were no differences in the prevalence of ERP between ethnicities [68]. The same authors reported proctogram findings in 727 patients, highlighting that the incidence of grade III–V rectal prolapse (high grade RI and ERP) was not significantly associated with ethnicity. However, in an ultrasonic assessment of pelvic floor mobility (including the posterior compartment), Asian women had significantly less mobility than white women [69].

### Which genetic variants affecting connective tissue genes have been found to be associated with rectal prolapse?

No candidate gene studies or studies using linkage analysis were identified in the context of rectal prolapse. A single candidate gene study found that variants in *GSTM1* were significantly more common in patients with anorectal outlet obstruction than in those without, though only 6/40 of these patients had rectal prolapse [70].

However, six studies reported results from whole exome or whole genome sequencing (or extensive genotyping) in patients with rectal prolapse. A novel *EFEMP1* (fibulin‐3) variant in siblings with phenotypic traits of connective tissue disease (Marfanoid appearance, joint laxity, multiple hernias and rectal prolapse) [71] and a novel de novo variant in *COLGALT2* (GLT25 D2 enzyme) in a patient with mucosal prolapse, POP and radiological evidence of vascular changes [72] were identified. Further novel variants were reported in connective tissue genes in patients with rectal prolapse as a feature of cutis laxa (*FBLN5*) [73], clEDS (*TNXB*) [74] and arterial tortuosity syndrome (*SLC2A10*) [75]. Finally a variant in *COL1A1* (Collagen Type I Alpha 1 Chain) in a patient with hypermobility and descending perineum syndrome was identified [76]. No characterisation of polygenic risk (via the use of genome wide association studies) was identified.

## DISCUSSION

### Narrative synthesis

The high incidence of ERP in patients with HDCTs and hypermobility supports the conclusion that genetic connective tissue defects predispose to the development of rectal prolapse. Furthermore, concurrent HDCTs or hypermobility appear to correlate with a younger onset and poorer post‐operative outcomes.

In order to capture the full breadth of the literature on this topic, a broad range of search terms were chosen including a comprehensive list of terms that may be used to define joint hypermobility (as detailed in Figure S1). It should be noted that the variability in the way in which hypermobility is defined and quantified means that elucidating the true magnitude of the association with rectal prolapse is challenging.

A large proportion of the evidence relates to patients with EDS. The genetic basis of the most common clinical subtype of EDS (hypermobile EDS) and generalised hypermobility remains unknown. The pathophysiological pathway that may predispose to the development of rectal prolapse therefore remains elusive.

Histological analysis of pelvic floor tissues has demonstrated that rectal prolapse is associated with abnormal rectal collagen and elastin content, abnormal fibre morphology and increased MMP1 expression. It is also associated with reduced myofibroblast density in males and parous females, and with increased cellular proliferation in females. This may simply reflect tissue damage and compensatory changes in response to trauma caused by the prolapse. However, a systemic connective tissue disorder is a plausible alternative explanation. Higher dermal elastin and mechanical tissue compliance in skin samples from patients with rectal prolapse provide the most compelling evidence of a systemic connective tissue disorder.

The significance of differences identified in age and gender subgroup analyses is also uncertain. Thinner collagen layers in male and nulliparous females with ERP compared to parous females may reflect childbirth‐induced trauma or could be indicative of a systemic reduction in effective collagen synthesis in subgroups (males and nulliparous females) who lack other risk factors for ERP. Evidence of reduced Fibulin‐5 expression in young males with rectal prolapse, compared to age‐matched controls, which was not replicated in other subgroups, provides support for the latter.

The paucity of familial aggregation studies limits our ability to characterise the pattern of inheritance or the penetrance in ERP. Rectal prolapse may, in some cases, be a manifestation of an undefined monogenic connective tissue disease. Family studies would help establish a Mendelian inheritance pattern in this case. In other cases, it is possible that the genetic contribution to disease risk is of a complex, polygenic nature. Familial aggregation studies may provide evidence of clustering to support this, whereas twin studies could establish the magnitude of the genetic contribution. Establishing whether familial clustering of rectal prolapse is associated with other phenotypic features such as generalised hypermobility would support the hypothesis that variants in connective tissue genes are implicated. Higher pelvic floor mobility in white women compared to Asian women may reflect a genetic vulnerability. This, however, is not supported by the limited epidemiological data.

The use of next‐generation sequencing has allowed identification of pathogenic variants in connective tissue‐related genes in patients with rectal prolapse with either recognised HDCTs or undefined ‘connective tissue phenotypes’. The proteins encoded by genes in which these variants have been identified play important roles in elastic fibre maintenance (*EFEMP1, FBLN5*) and collagen fibril organisation (*TNXB, COLGALT2*). This provides an intriguing insight into a potential genotype–phenotype relationship, accounting for how defective extracellular matrix assembly may result in the clinical manifestation. Genome sequencing in a greater number of patients with rectal prolapse, particularly those with a more ‘extreme phenotype’, may provide stronger evidence for the role of such variants in its pathogenesis.

Rectal prolapse appears to be a feature of some monogenic connective tissue diseases, but this is likely to only represent a small proportion of cases. The genetic contribution to the aetiology of rectal prolapse in most cases is likely to be polygenic. Characterisation of the polygenic risk profile of rectal prolapse would best be addressed with the utilisation of large databases of genotyped individuals (such as the UK Biobank) to carry out a genome wide association study (GWAS). Such an approach would allow the identification of the cumulative impact of a larger number of genetic variants.

### Insights from genetic research in POP

Like rectal prolapse, POP is associated with joint hypermobility and HDCTs [77, 78, 79, 80]. A 2022 systematic review and meta‐analysis (including 18 studies) identified an odds ratio of 2.64 for developing POP in those with a family history of POP compared to those without [81]. Utilising a cohort of over 16,000 individuals from the Swedish Twin Registry, 43% of the risk of POP can be attributed to genetics [82].

A 2021 systematic review and meta‐analysis of candidate gene studies, incorporating 53 studies identified variants in four genes (*FLBN5, COL1A1, PGR* and *ESR1*) with a significant association with POP in pooled analysis. Fibulin‐5 (*FLBN5*) and Collagen Type I Alpha 1 (*COL1A1*) are important extracellular matrix proteins.

A 2022 meta‐analysis of GWAS from four European biobanks (incorporating over 500,000 participants) identified 30 genetic variants reaching genome‐wide significant association with POP. These included variants close to *LOXL1, EFEMP1, CHRDL2, CRISPLD2* and *ADAMTS5* genes, all of which are implicated in extracellular matrix synthesis and regulation [15]. Pairwise correlation with other GWAS studies demonstrated that the genetic risk for POP correlated with that of diverticular disease and hiatus hernia. This supports the conclusion of a unifying genetic predisposition for such conditions with underlying abnormalities of connective tissue.

Although a degree of mutual pathophysiology between ERP and POP is highly likely, caution should be exercised in directly extrapolating from these findings. The distinct anatomy and physiology of the anus and rectum demand further research specifically in this area.

## CONCLUSION

Rectal prolapse is an anatomical abnormality, resulting from failure of the pelvic floor and rectal support structures. Its aetiology is multifactorial and heterogeneous. Connective tissues are integral for the pelvic floor to act as a mechanical support structure. Ineffective connective tissues, whether due to deleterious genetic variants or secondary to other factors, are likely to contribute to the development of rectal prolapse.

Further research should focus on establishing the pattern and extent of heritability in rectal prolapse. This should include collecting detailed family history data from large cohorts and the use of twin databases. Furthermore, whole genome or exome sequencing of individuals with an extreme phenotype should be utilised to identify potentially pathogenic genetic variants. The significance of such variants in patients with rectal prolapse could then be interrogated by means of a genetic association study. Finally, GWAS, using resources such as the UK Biobank, should be undertaken to characterise the nature of polygenic risk in rectal prolapse.

## AUTHOR CONTRIBUTIONS

**Matthew Davenport:** Conceptualization; methodology; formal analysis; writing – original draft; writing – review and editing; data curation. **Alexander O'Connor:** Conceptualization; methodology; data curation; validation; supervision; writing – review and editing. **William G. Newman:** Conceptualization; supervision; writing – review and editing; methodology. **Andrew P. Morris:** Conceptualization; methodology; writing – review and editing; supervision. **Abhiram Sharma:** Conceptualization; methodology; supervision; writing – review and editing. **Gemma Faulkner:** Conceptualization; methodology; supervision; writing – review and editing. **Dipesh H. Vasant:** Conceptualization; methodology; supervision; writing – review and editing. **John McLaughlin:** Conceptualization; methodology; supervision; writing – review and editing. **Edward Kiff:** Conceptualization; methodology; supervision; writing – review and editing. **Karen Telford:** Conceptualization; methodology; supervision; writing – review and editing.

## FUNDING INFORMATION

MD received funding from GUTS UK & Royal College of Surgeons (Edinburgh) for research into the genetic basis of rectal prolapse. WGN is supported by the NIHR Manchester BRC (NIHR203308).

## CONFLICT OF INTEREST STATEMENT

The authors declare that there is no conflict of interest.

## ETHICAL APPROVAL

N/A.

## PATIENT CONSENT STATEMENT

N/A.

## PERMISSION TO REPRODUCE MATERIAL FROM OTHER SOURCES

N/A.

## CLINICAL TRIAL REGISTRATION

N/A.

## Supporting information


**Figure S1.** Literature search methodology.

**Figure S2.** Data charting items for full‐text screening.

**Data S1.** Preferred reporting items for systematic reviews and meta‐analyses extension for scoping reviews (PRISMA‐ScR) checklist.

## Data Availability

Data sharing not applicable to this article as no new datasets were generated for this scoping review.
