# Supplementary material for: Connective tissue and genetics in the pathogenesis of rectal prolapse: A scoping review
Source: Colorectal Dis. 2026 Sep 21;28(9):e70628. doi: 10.1111/codi.70628 (PMC13591292; doi:10.1111/codi.70628)
Supplement: Supplementary file 1 [file CODI-28-0-s001.docx]

**Supplementary figure 2**

Data charting items for full text screening

| Study reference |  |
| --- | --- |
| resource type (peer reviewed manuscript, conference proceedings) |  |
| Country |  |
| Study design |  |
| Study design additional detail |  |
| Participant characteristics |  |
| Specific concept examined |  |
| Key results |  |
| Include/Exclude |  |
| reason for exclusion |  |
| Additional information |  |
