# Supplementary material for: Connective tissue and genetics in the pathogenesis of rectal prolapse: A scoping review
Source: Colorectal Dis. 2026 Sep 21;28(9):e70628. doi: 10.1111/codi.70628 (PMC13591292; doi:10.1111/codi.70628)
Supplement: Supplementary file 3 [file CODI-28-0-s003.docx]

**Supplementary figure 1**

Literature search methodology

I. **Medline search**

**Initial search: October 2025**

Ovid MEDLINE(R) ALL <1946 to October 14, 2025>

1	(Rectal prolapse or Rectal procidentia or Rectal intussusception or Pelvic Organ Prolapse or Genito-urinary prolapse or Rectocele or Cystocele or Uterine prolapse or Vaginal vault prolapse or Enterocele).ti,ab.	13889

2	Rectal Prolapse/	3251

3	Uterine Prolapse/ or Pelvic Organ Prolapse/	11746

4	1 or 2 or 3	19671

5	("Hereditary connective tissue disorder" or "Hereditary connective tissue disorders" or "Hereditary Disorder of Connective Tissue" or "Hereditary Disorders of Connective Tissue" or "Heritable disorder of connective tissue" or "Heritable disorders of connective tissue" or "Heritable connective tissue disorder" or "Heritable connective tissue disorders" or "Arterial tortuosity syndrome" or "Congenital contractural arachnodactyly" or "Beals–Hecht syndrome" or "Cutis laxa" or "Ehlers-Danlos syndrome" or "Ehlers Danlos" or "Familial thoracic aortic aneurysm" or "Familial thoracic aortic aneurysm and dissection" or "Familial thoracic dissection" or "familial TAAD" or "Homocystinuria" or "LH3 deficiency syndrome" or "BCARD syndrome" or "Loeys-Dietz syndrome" or "Lujan syndrome" or "MACS syndrome" or "RIN2 syndrome" or "Marfan syndrome" or "Marfan's" or "Marfan-like" or "Marfanoid" or "MASS phenotype" or "MASS syndrome" or "Osteogenesis imperfecta" or "Shprintzen-Goldberg syndrome" or "Stickler syndrome" or "Weill-Marchesani syndrome" or "Generalised Joint Hypermobility" or "Hypermobility spectrum disorder" or "Hypermobility" or "Joint laxity" or "Beighton").mp.	31355

6	("Connective tissue" or "Extracellular matrix" or "Collagen" or "Crosslink" or "crosslinking" or "Fibrillin" or "Fibrillins" or "Fibulin" or "Fibulins" or "Elastin" or "Elastins" or "Fibronectin" or "Tenascin" or "Tenascins" or "lysyl hydroxylase" or "lysyl hydroxylases" or "Proteoglycan" or "Proteoglycans" or "Integrin" or "integrins" or "Laminin" or "Laminins" or "Matrix metalloproteinase" or "Matrix metalloproteinases" or "MMP" or "MMPs" or "Tissue Inhibitors of Metalloproteinase" or "Tissue Inhibitors of Metalloproteinases" or "TIMP" or "TIMPs" or "serine protease" or "serine proteases" or "expression" or "histology" or "immunohistochemistry" or "immunofluorescence" or "in vitro" or "ex vivo" or "Transcriptomics" or "sequencing" or "Proteomics" or "Muti-omics" or "Mass spectrometry" or "CRISPR" or "Heritability" or "Twin" or "Family study" or "family linkage" or "family linkage study" or "Pedigree" or "Inheritance" or "Segregation" or "Co-segregation" or "Ethnicity" or "Ethnic" or "Ancestry" or "genes" or "Candidate gene" or "Polymorphism" or "Polymorphisms" or "Variant" or "Variants" or "Mutation" or "Mutations" or "SNP" or "SNPs" or "Single nucleotide polymorphism" or "Single nucleotide polymorphisms" or "Copy number variant" or "Copy number variants" or "Structural variant" or "Structural variants" or "Genotype" or "genotyping" or "Whole genome" or "Whole exome" or "Genome wide association study" or "GWAS" or "Biobank" or "Mendelian Randomization").mp.	8366921

7	5 or 6	8384739

8	4 and 7	1959

9	limit 8 to english language	1815

**Search update: March 2026**

Ovid MEDLINE(R) ALL <1946 to March 26, 2026>

1	(Rectal prolapse or Rectal procidentia or Rectal intussusception or Pelvic Organ Prolapse or Genito-urinary prolapse or Rectocele or Cystocele or Uterine prolapse or Vaginal vault prolapse or Enterocele).ti,ab.	14269

2	Rectal Prolapse/	3273

3	Uterine Prolapse/ or Pelvic Organ Prolapse/	11947

4	1 or 2 or 3	20078

5	("Hereditary connective tissue disorder" or "Hereditary connective tissue disorders" or "Hereditary Disorder of Connective Tissue" or "Hereditary Disorders of Connective Tissue" or "Heritable disorder of connective tissue" or "Heritable disorders of connective tissue" or "Heritable connective tissue disorder" or "Heritable connective tissue disorders" or "Arterial tortuosity syndrome" or "Congenital contractural arachnodactyly" or "Beals–Hecht syndrome" or "Cutis laxa" or "Ehlers-Danlos syndrome" or "Ehlers Danlos" or "Familial thoracic aortic aneurysm" or "Familial thoracic aortic aneurysm and dissection" or "Familial thoracic dissection" or "familial TAAD" or "Homocystinuria" or "LH3 deficiency syndrome" or "BCARD syndrome" or "Loeys-Dietz syndrome" or "Lujan syndrome" or "MACS syndrome" or "RIN2 syndrome" or "Marfan syndrome" or "Marfan's" or "Marfan-like" or "Marfanoid" or "MASS phenotype" or "MASS syndrome" or "Osteogenesis imperfecta" or "Shprintzen-Goldberg syndrome" or "Stickler syndrome" or "Weill-Marchesani syndrome" or "Generalised Joint Hypermobility" or "Hypermobility spectrum disorder" or "Hypermobility" or "Joint laxity" or "Beighton").mp.	31978

6	("Connective tissue" or "Extracellular matrix" or "Collagen" or "Crosslink" or "crosslinking" or "Fibrillin" or "Fibrillins" or "Fibulin" or "Fibulins" or "Elastin" or "Elastins" or "Fibronectin" or "Tenascin" or "Tenascins" or "lysyl hydroxylase" or "lysyl hydroxylases" or "Proteoglycan" or "Proteoglycans" or "Integrin" or "integrins" or "Laminin" or "Laminins" or "Matrix metalloproteinase" or "Matrix metalloproteinases" or "MMP" or "MMPs" or "Tissue Inhibitors of Metalloproteinase" or "Tissue Inhibitors of Metalloproteinases" or "TIMP" or "TIMPs" or "serine protease" or "serine proteases" or "expression" or "histology" or "immunohistochemistry" or "immunofluorescence" or "in vitro" or "ex vivo" or "Transcriptomics" or "sequencing" or "Proteomics" or "Muti-omics" or "Mass spectrometry" or "CRISPR" or "Heritability" or "Twin" or "Family study" or "family linkage" or "family linkage study" or "Pedigree" or "Inheritance" or "Segregation" or "Co-segregation" or "Ethnicity" or "Ethnic" or "Ancestry" or "genes" or "Candidate gene" or "Polymorphism" or "Polymorphisms" or "Variant" or "Variants" or "Mutation" or "Mutations" or "SNP" or "SNPs" or "Single nucleotide polymorphism" or "Single nucleotide polymorphisms" or "Copy number variant" or "Copy number variants" or "Structural variant" or "Structural variants" or "Genotype" or "genotyping" or "Whole genome" or "Whole exome" or "Genome wide association study" or "GWAS" or "Biobank" or "Mendelian Randomization").mp.	8555917

7	5 or 6	8574060

8	4 and 7	1993

9	limit 8 to english language	1849

10	from 9 keep 1-1849	1849

11	limit 9 to dt=20251014-20260327	33

II. **Embase search**

**Initial search: October 2025**

Embase <1980 to 2025 Week 41>

1	(Rectal prolapse or Rectal procidentia or Rectal intussusception or Pelvic Organ Prolapse or Genito-urinary prolapse or Rectocele or Cystocele or Uterine prolapse or Vaginal vault prolapse or Enterocele).ti,ab.	23987

2	Rectal Prolapse/	6031

3	Uterine Prolapse/ or Pelvic Organ Prolapse/	23963

4	1 or 2 or 3	34432

5	("Hereditary connective tissue disorder" or "Hereditary connective tissue disorders" or "Hereditary Disorder of Connective Tissue" or "Hereditary Disorders of Connective Tissue" or "Heritable disorder of connective tissue" or "Heritable disorders of connective tissue" or "Heritable connective tissue disorder" or "Heritable connective tissue disorders" or "Arterial tortuosity syndrome" or "Congenital contractural arachnodactyly" or "Beals–Hecht syndrome" or "Cutis laxa" or "Ehlers-Danlos syndrome" or "Ehlers Danlos" or "Familial thoracic aortic aneurysm" or "Familial thoracic aortic aneurysm and dissection" or "Familial thoracic dissection" or "familial TAAD" or "Homocystinuria" or "LH3 deficiency syndrome" or "BCARD syndrome" or "Loeys-Dietz syndrome" or "Lujan syndrome" or "MACS syndrome" or "RIN2 syndrome" or "Marfan syndrome" or "Marfan's" or "Marfan-like" or "Marfanoid" or "MASS phenotype" or "MASS syndrome" or "Osteogenesis imperfecta" or "Shprintzen-Goldberg syndrome" or "Stickler syndrome" or "Weill-Marchesani syndrome" or "Generalised Joint Hypermobility" or "Hypermobility spectrum disorder" or "Hypermobility" or "Joint laxity" or "Beighton").mp.	47646

6	("Connective tissue" or "Extracellular matrix" or "Collagen" or "Crosslink" or "crosslinking" or "Fibrillin" or "Fibrillins" or "Fibulin" or "Fibulins" or "Elastin" or "Elastins" or "Fibronectin" or "Tenascin" or "Tenascins" or "lysyl hydroxylase" or "lysyl hydroxylases" or "Proteoglycan" or "Proteoglycans" or "Integrin" or "integrins" or "Laminin" or "Laminins" or "Matrix metalloproteinase" or "Matrix metalloproteinases" or "MMP" or "MMPs" or "Tissue Inhibitors of Metalloproteinase" or "Tissue Inhibitors of Metalloproteinases" or "TIMP" or "TIMPs" or "serine protease" or "serine proteases" or "expression" or "histology" or "immunohistochemistry" or "immunofluorescence" or "in vitro" or "ex vivo" or "Transcriptomics" or "sequencing" or "Proteomics" or "Muti-omics" or "Mass spectrometry" or "CRISPR" or "Heritability" or "Twin" or "Family study" or "family linkage" or "family linkage study" or "Pedigree" or "Inheritance" or "Segregation" or "Co-segregation" or "Ethnicity" or "Ethnic" or "Ancestry" or "genes" or "Candidate gene" or "Polymorphism" or "Polymorphisms" or "Variant" or "Variants" or "Mutation" or "Mutations" or "SNP" or "SNPs" or "Single nucleotide polymorphism" or "Single nucleotide polymorphisms" or "Copy number variant" or "Copy number variants" or "Structural variant" or "Structural variants" or "Genotype" or "genotyping" or "Whole genome" or "Whole exome" or "Genome wide association study" or "GWAS" or "Biobank" or "Mendelian Randomization").mp.	10963956

7	5 or 6	10988993

8	4 and 7	4207

9	limit 8 to english language	3999

**Search update: March 2026**

Embase <1980 to 2026 Week 12>

1	(Rectal prolapse or Rectal procidentia or Rectal intussusception or Pelvic Organ Prolapse or Genito-urinary prolapse or Rectocele or Cystocele or Uterine prolapse or Vaginal vault prolapse or Enterocele).ti,ab.	24784

2	Rectal Prolapse/	6212

3	Uterine Prolapse/ or Pelvic Organ Prolapse/	24679

4	1 or 2 or 3	35412

5	("Hereditary connective tissue disorder" or "Hereditary connective tissue disorders" or "Hereditary Disorder of Connective Tissue" or "Hereditary Disorders of Connective Tissue" or "Heritable disorder of connective tissue" or "Heritable disorders of connective tissue" or "Heritable connective tissue disorder" or "Heritable connective tissue disorders" or "Arterial tortuosity syndrome" or "Congenital contractural arachnodactyly" or "Beals–Hecht syndrome" or "Cutis laxa" or "Ehlers-Danlos syndrome" or "Ehlers Danlos" or "Familial thoracic aortic aneurysm" or "Familial thoracic aortic aneurysm and dissection" or "Familial thoracic dissection" or "familial TAAD" or "Homocystinuria" or "LH3 deficiency syndrome" or "BCARD syndrome" or "Loeys-Dietz syndrome" or "Lujan syndrome" or "MACS syndrome" or "RIN2 syndrome" or "Marfan syndrome" or "Marfan's" or "Marfan-like" or "Marfanoid" or "MASS phenotype" or "MASS syndrome" or "Osteogenesis imperfecta" or "Shprintzen-Goldberg syndrome" or "Stickler syndrome" or "Weill-Marchesani syndrome" or "Generalised Joint Hypermobility" or "Hypermobility spectrum disorder" or "Hypermobility" or "Joint laxity" or "Beighton").mp.	49096

6	("Connective tissue" or "Extracellular matrix" or "Collagen" or "Crosslink" or "crosslinking" or "Fibrillin" or "Fibrillins" or "Fibulin" or "Fibulins" or "Elastin" or "Elastins" or "Fibronectin" or "Tenascin" or "Tenascins" or "lysyl hydroxylase" or "lysyl hydroxylases" or "Proteoglycan" or "Proteoglycans" or "Integrin" or "integrins" or "Laminin" or "Laminins" or "Matrix metalloproteinase" or "Matrix metalloproteinases" or "MMP" or "MMPs" or "Tissue Inhibitors of Metalloproteinase" or "Tissue Inhibitors of Metalloproteinases" or "TIMP" or "TIMPs" or "serine protease" or "serine proteases" or "expression" or "histology" or "immunohistochemistry" or "immunofluorescence" or "in vitro" or "ex vivo" or "Transcriptomics" or "sequencing" or "Proteomics" or "Muti-omics" or "Mass spectrometry" or "CRISPR" or "Heritability" or "Twin" or "Family study" or "family linkage" or "family linkage study" or "Pedigree" or "Inheritance" or "Segregation" or "Co-segregation" or "Ethnicity" or "Ethnic" or "Ancestry" or "genes" or "Candidate gene" or "Polymorphism" or "Polymorphisms" or "Variant" or "Variants" or "Mutation" or "Mutations" or "SNP" or "SNPs" or "Single nucleotide polymorphism" or "Single nucleotide polymorphisms" or "Copy number variant" or "Copy number variants" or "Structural variant" or "Structural variants" or "Genotype" or "genotyping" or "Whole genome" or "Whole exome" or "Genome wide association study" or "GWAS" or "Biobank" or "Mendelian Randomization").mp.	11272481

7	5 or 6	11298173

8	4 and 7	4350

9	limit 8 to english language	4140

10	9	4140

11	limit 10 to yr="2025 -Current"	243

III. **CINAHL search**

**Initial Search: October 2025:**

((("Hereditary connective tissue disorder" or "Hereditary connective tissue disorders" or "Hereditary Disorder of Connective Tissue" or "Hereditary Disorders of Connective Tissue" or "Heritable disorder of connective tissue" or "Heritable disorders of connective tissue" or "Heritable connective tissue disorder" or "Heritable connective tissue disorders" or "Arterial tortuosity syndrome" or "Congenital contractural arachnodactyly" or "Beals–Hecht syndrome" or "Cutis laxa" or "Ehlers-Danlos syndrome" or "Ehlers Danlos" or "Familial thoracic aortic aneurysm" or "Familial thoracic aortic aneurysm and dissection" or "Familial thoracic dissection" or "familial TAAD" or "Homocystinuria" or "LH3 deficiency syndrome" or "BCARD syndrome" or "Loeys-Dietz syndrome" or "Lujan syndrome" or "MACS syndrome" or "RIN2 syndrome" or "Marfan syndrome" or "Marfan's" or "Marfan-like" or "Marfanoid" or "MASS phenotype" or "MASS syndrome" or "Osteogenesis imperfecta" or "Shprintzen-Goldberg syndrome" or "Stickler syndrome" or "Weill-Marchesani syndrome" or "Generalised Joint Hypermobility" or "Hypermobility spectrum disorder" or "Hypermobility" or "Joint laxity" or "Beighton")) OR (("Connective tissue" or "Extracellular matrix" or "Collagen" or "Crosslink" or "crosslinking" or "Fibrillin" or "Fibrillins" or "Fibulin" or "Fibulins" or "Elastin" or "Elastins" or "Fibronectin" or "Tenascin" or "Tenascins" or "lysyl hydroxylase" or "lysyl hydroxylases" or "Proteoglycan" or "Proteoglycans" or "Integrin" or "integrins" or "Laminin" or "Laminins" or "Matrix metalloproteinase" or "Matrix metalloproteinases" or "MMP" or "MMPs" or "Tissue Inhibitors of Metalloproteinase" or "Tissue Inhibitors of Metalloproteinases" or "TIMP" or "TIMPs" or "serine protease" or "serine proteases" or "expression" or "histology" or "immunohistochemistry" or "immunofluorescence" or "in vitro" or "ex vivo" or "Transcriptomics" or "sequencing" or "Proteomics" or "Muti-omics" or "Mass spectrometry" or "CRISPR" or "Heritability" or "Twin" or "Family study" or "family linkage" or "family linkage study" or "Pedigree" or "Inheritance" or "Segregation" or "Co-segregation" or "Ethnicity" or "Ethnic" or "Ancestry" or "genes" or "Candidate gene" or "Polymorphism" or "Polymorphisms" or "Variant" or "Variants" or "Mutation" or "Mutations" or "SNP" or "SNPs" or "Single nucleotide polymorphism" or "Single nucleotide polymorphisms" or "Copy number variant" or "Copy number variants" or "Structural variant" or "Structural variants" or "Genotype" or "genotyping" or "Whole genome" or "Whole exome" or "Genome wide association study" or "GWAS" or "Biobank" or "Mendelian Randomization"))) AND (XB ((Rectal prolapse or Rectal procidentia or Rectal intussusception or Pelvic Organ Prolapse or Genito-urinary prolapse or Rectocele or Cystocele or Uterine prolapse or Vaginal vault prolapse or Enterocele)) OR MH "Rectal Prolapse" OR (MH "Pelvic Organ Prolapse" OR MH "Uterine Prolapse"))

Results

464

Filters

English Language

**Search update: March 2026**

((("Hereditary connective tissue disorder" or "Hereditary connective tissue disorders" or "Hereditary Disorder of Connective Tissue" or "Hereditary Disorders of Connective Tissue" or "Heritable disorder of connective tissue" or "Heritable disorders of connective tissue" or "Heritable connective tissue disorder" or "Heritable connective tissue disorders" or "Arterial tortuosity syndrome" or "Congenital contractural arachnodactyly" or "Beals–Hecht syndrome" or "Cutis laxa" or "Ehlers-Danlos syndrome" or "Ehlers Danlos" or "Familial thoracic aortic aneurysm" or "Familial thoracic aortic aneurysm and dissection" or "Familial thoracic dissection" or "familial TAAD" or "Homocystinuria" or "LH3 deficiency syndrome" or "BCARD syndrome" or "Loeys-Dietz syndrome" or "Lujan syndrome" or "MACS syndrome" or "RIN2 syndrome" or "Marfan syndrome" or "Marfan's" or "Marfan-like" or "Marfanoid" or "MASS phenotype" or "MASS syndrome" or "Osteogenesis imperfecta" or "Shprintzen-Goldberg syndrome" or "Stickler syndrome" or "Weill-Marchesani syndrome" or "Generalised Joint Hypermobility" or "Hypermobility spectrum disorder" or "Hypermobility" or "Joint laxity" or "Beighton")) OR (("Connective tissue" or "Extracellular matrix" or "Collagen" or "Crosslink" or "crosslinking" or "Fibrillin" or "Fibrillins" or "Fibulin" or "Fibulins" or "Elastin" or "Elastins" or "Fibronectin" or "Tenascin" or "Tenascins" or "lysyl hydroxylase" or "lysyl hydroxylases" or "Proteoglycan" or "Proteoglycans" or "Integrin" or "integrins" or "Laminin" or "Laminins" or "Matrix metalloproteinase" or "Matrix metalloproteinases" or "MMP" or "MMPs" or "Tissue Inhibitors of Metalloproteinase" or "Tissue Inhibitors of Metalloproteinases" or "TIMP" or "TIMPs" or "serine protease" or "serine proteases" or "expression" or "histology" or "immunohistochemistry" or "immunofluorescence" or "in vitro" or "ex vivo" or "Transcriptomics" or "sequencing" or "Proteomics" or "Muti-omics" or "Mass spectrometry" or "CRISPR" or "Heritability" or "Twin" or "Family study" or "family linkage" or "family linkage study" or "Pedigree" or "Inheritance" or "Segregation" or "Co-segregation" or "Ethnicity" or "Ethnic" or "Ancestry" or "genes" or "Candidate gene" or "Polymorphism" or "Polymorphisms" or "Variant" or "Variants" or "Mutation" or "Mutations" or "SNP" or "SNPs" or "Single nucleotide polymorphism" or "Single nucleotide polymorphisms" or "Copy number variant" or "Copy number variants" or "Structural variant" or "Structural variants" or "Genotype" or "genotyping" or "Whole genome" or "Whole exome" or "Genome wide association study" or "GWAS" or "Biobank" or "Mendelian Randomization"))) AND (XB ((Rectal prolapse or Rectal procidentia or Rectal intussusception or Pelvic Organ Prolapse or Genito-urinary prolapse or Rectocele or Cystocele or Uterine prolapse or Vaginal vault prolapse or Enterocele)) OR MH "Rectal Prolapse" OR (MH "Pelvic Organ Prolapse" OR MH "Uterine Prolapse"))

(Limited to Oct 2025 to Mar 2026)

IV. **Cochrane Library:**

**Initial search: October 2025**

Date Run:	16/10/2025 06:43:22

ID	Search	Hits

#1	(Rectal prolapse or Rectal procidentia or Rectal intussusception or Pelvic Organ Prolapse or Genito-urinary prolapse or Rectocele or Cystocele or Uterine prolapse or Vaginal vault prolapse or Enterocele):ti,ab,kw	2973

#2	MeSH descriptor: [Rectal Prolapse] explode all trees	102

#3	MeSH descriptor: [Pelvic Organ Prolapse] explode all trees	1021

#4	MeSH descriptor: [Uterine Prolapse] explode all trees	321

#5	#1 OR #2 OR #3 OR #4	2973

#6	(("Hereditary connective tissue disorder" or "Hereditary connective tissue disorders" or "Hereditary Disorder of Connective Tissue" or "Hereditary Disorders of Connective Tissue" or "Heritable disorder of connective tissue" or "Heritable disorders of connective tissue" or "Heritable connective tissue disorder" or "Heritable connective tissue disorders" or "Arterial tortuosity syndrome" or "Congenital contractural arachnodactyly" or "Beals–Hecht syndrome" or "Cutis laxa" or "Ehlers-Danlos syndrome" or "Ehlers Danlos" or "Familial thoracic aortic aneurysm" or "Familial thoracic aortic aneurysm and dissection" or "Familial thoracic dissection" or "familial TAAD" or "Homocystinuria" or "LH3 deficiency syndrome" or "BCARD syndrome" or "Loeys-Dietz syndrome" or "Lujan syndrome" or "MACS syndrome" or "RIN2 syndrome" or "Marfan syndrome" or "Marfan's" or "Marfan-like" or "Marfanoid" or "MASS phenotype" or "MASS syndrome" or "Osteogenesis imperfecta" or "Shprintzen-Goldberg syndrome" or "Stickler syndrome" or "Weill-Marchesani syndrome" or "Generalised Joint Hypermobility" or "Hypermobility spectrum disorder" or "Hypermobility" or "Joint laxity" or "Beighton")):ti,ab,kw	894

#7	(("Connective tissue" or "Extracellular matrix" or "Collagen" or "Crosslink" or "crosslinking" or "Fibrillin" or "Fibrillins" or "Fibulin" or "Fibulins" or "Elastin" or "Elastins" or "Fibronectin" or "Tenascin" or "Tenascins" or "lysyl hydroxylase" or "lysyl hydroxylases" or "Proteoglycan" or "Proteoglycans" or "Integrin" or "integrins" or "Laminin" or "Laminins" or "Matrix metalloproteinase" or "Matrix metalloproteinases" or "MMP" or "MMPs" or "Tissue Inhibitors of Metalloproteinase" or "Tissue Inhibitors of Metalloproteinases" or "TIMP" or "TIMPs" or "serine protease" or "serine proteases" or "expression" or "histology" or "immunohistochemistry" or "immunofluorescence" or "in vitro" or "ex vivo" or "Transcriptomics" or "sequencing" or "Proteomics" or "Muti-omics" or "Mass spectrometry" or "CRISPR" or "Heritability" or "Twin" or "Family study" or "family linkage" or "family linkage study" or "Pedigree" or "Inheritance" or "Segregation" or "Co-segregation" or "Ethnicity" or "Ethnic" or "Ancestry" or "genes" or "Candidate gene" or "Polymorphism" or "Polymorphisms" or "Variant" or "Variants" or "Mutation" or "Mutations" or "SNP" or "SNPs" or "Single nucleotide polymorphism" or "Single nucleotide polymorphisms" or "Copy number variant" or "Copy number variants" or "Structural variant" or "Structural variants" or "Genotype" or "genotyping" or "Whole genome" or "Whole exome" or "Genome wide association study" or "GWAS" or "Biobank" or "Mendelian Randomization")):ti,ab,kw	182376

#8	#6 OR #7	183096

#9	#5 AND #8	174

Limited to English language: 166

**Search update: March 2026**

Date Run:	27/03/2026 12:52:36

ID	Search	Hits

#1	(Rectal prolapse or Rectal procidentia or Rectal intussusception or Pelvic Organ Prolapse or Genito-urinary prolapse or Rectocele or Cystocele or Uterine prolapse or Vaginal vault prolapse or Enterocele):ti,ab,kw	3058

#2	MeSH descriptor: [Rectal Prolapse] explode all trees	103

#3	MeSH descriptor: [Pelvic Organ Prolapse] explode all trees	1040

#4	MeSH descriptor: [Uterine Prolapse] explode all trees	321

#5	#1 OR #2 OR #3 OR #4	3058

#6	(("Hereditary connective tissue disorder" or "Hereditary connective tissue disorders" or "Hereditary Disorder of Connective Tissue" or "Hereditary Disorders of Connective Tissue" or "Heritable disorder of connective tissue" or "Heritable disorders of connective tissue" or "Heritable connective tissue disorder" or "Heritable connective tissue disorders" or "Arterial tortuosity syndrome" or "Congenital contractural arachnodactyly" or "Beals–Hecht syndrome" or "Cutis laxa" or "Ehlers-Danlos syndrome" or "Ehlers Danlos" or "Familial thoracic aortic aneurysm" or "Familial thoracic aortic aneurysm and dissection" or "Familial thoracic dissection" or "familial TAAD" or "Homocystinuria" or "LH3 deficiency syndrome" or "BCARD syndrome" or "Loeys-Dietz syndrome" or "Lujan syndrome" or "MACS syndrome" or "RIN2 syndrome" or "Marfan syndrome" or "Marfan's" or "Marfan-like" or "Marfanoid" or "MASS phenotype" or "MASS syndrome" or "Osteogenesis imperfecta" or "Shprintzen-Goldberg syndrome" or "Stickler syndrome" or "Weill-Marchesani syndrome" or "Generalised Joint Hypermobility" or "Hypermobility spectrum disorder" or "Hypermobility" or "Joint laxity" or "Beighton")):ti,ab,kw	919

#7	(("Connective tissue" or "Extracellular matrix" or "Collagen" or "Crosslink" or "crosslinking" or "Fibrillin" or "Fibrillins" or "Fibulin" or "Fibulins" or "Elastin" or "Elastins" or "Fibronectin" or "Tenascin" or "Tenascins" or "lysyl hydroxylase" or "lysyl hydroxylases" or "Proteoglycan" or "Proteoglycans" or "Integrin" or "integrins" or "Laminin" or "Laminins" or "Matrix metalloproteinase" or "Matrix metalloproteinases" or "MMP" or "MMPs" or "Tissue Inhibitors of Metalloproteinase" or "Tissue Inhibitors of Metalloproteinases" or "TIMP" or "TIMPs" or "serine protease" or "serine proteases" or "expression" or "histology" or "immunohistochemistry" or "immunofluorescence" or "in vitro" or "ex vivo" or "Transcriptomics" or "sequencing" or "Proteomics" or "Muti-omics" or "Mass spectrometry" or "CRISPR" or "Heritability" or "Twin" or "Family study" or "family linkage" or "family linkage study" or "Pedigree" or "Inheritance" or "Segregation" or "Co-segregation" or "Ethnicity" or "Ethnic" or "Ancestry" or "genes" or "Candidate gene" or "Polymorphism" or "Polymorphisms" or "Variant" or "Variants" or "Mutation" or "Mutations" or "SNP" or "SNPs" or "Single nucleotide polymorphism" or "Single nucleotide polymorphisms" or "Copy number variant" or "Copy number variants" or "Structural variant" or "Structural variants" or "Genotype" or "genotyping" or "Whole genome" or "Whole exome" or "Genome wide association study" or "GWAS" or "Biobank" or "Mendelian Randomization")):ti,ab,kw	186988

#8	#6 OR #7	187728

#9	#5 AND #8 with Cochrane Library publication date Between Oct 2025 and Mar 2026	5
